# Geriatric intervention in elderly patients with hip fracture in an orthopedic ward

**DOI:** 10.5249/jivr.v4i2.96

**Published:** 2012-07

**Authors:** Merete Gregersen, Marianne Metz Mørch, Kjeld Hougaard, Else Marie Damsgaard

**Affiliations:** ^*a*^Departments of Geriatrics, Aarhus University Hospital, DK-8000 Aarhus, Denmark.; ^*b*^Departments of Orthopedic Surgery, Aarhus University Hospital, DK-8000 Aarhus, Denmark.

## Abstract

**Background:**

Hip fracture is a common cause of long hospital stay in the elderly. Approximately one third of these patients die within the first year. As a consequence geriatric and orthopedic collaboration (orthogeriatrics) has been organized in different ways. The aim of this study is to evaluate the efficiency of a multidisciplinary geriatric in-hospital intervention on patient outcome.

**Methods:**

A total of 495 elderly hip fracture patients consecutively admitted to orthopedic surgery, were followed. Data were based on medical records. The intervention group (n=233) was compared to a historical cohort group (n=262) receiving traditional orthopedic treatment. Intervention program was based on initial physical and mental screening and evaluation, geriatric-focused care, and early discharge planning. The intervention was provided by a multidisciplinary geriatric team. After discharge, follow-up home-visits by a physiotherapist were performed, except for patients discharged to nursing homes, due to a 24-hour staff and easy access to the GP.

**Results:**

Median length of stay was reduced from 15 to 13 days. More patients began treatment with calcium/vitamin-D and bisphosphonate (p=sig). There was no difference in hemoglobin variation between the time of admission and three to six months post admission, and no difference in three-month readmissions (odds ratio (OR) = 1.09 [95%CI: 0.71;1.67]). Discharge destination was unchanged (OR=0.93 [95%CI: 0.52; 1.65]). In-hospital mortality was 8% in the intervention group vs. 6% (p=0.48), in the control group. Three-month mortality was 16% in the intervention group vs. 15% (p=0.39), in the control group. In the intervention group, residents from nursing homes had a higher three-month mortality (OR=2.37 [95% CI: 0.99; 5.67]), and the risk of new fractures within two years decreased from 9.5% to 7.7%, though not statistically significant.

**Conclusion:**

Our study indicates that co-management of hip fracture patients by orthopedic surgeons and geriatricians may be associated with a reduction in length of hospital stay without negatively affecting major patient outcomes. The concept should be further developed particularly among the frail elderly.

## Introduction

In Denmark, annually, approximately 7,000 hip fractures occur among the 65+ -years old. The incidence is increasing in those aged 85 years or older.^1^ The elderly patient becomes weaker by hospitalization and surgery and often does not achieve the same level of functional ability as prior to their injury incident and subsequent hospitalization and surgery. Approximately one third of the patients with hip fracture die within the first year after surgery.^2^The existing literature suggests that there is a need to improve the efficiency and quality of hip fracture injury treatments and care of patients involved with such injuries. To this end, the geriatricians along with orthopedic surgeons have organized an effort toward forming so called “orthogeriatrics” collaboration in various countries.^3,4^Devas and his colleagues in Hastings, England, are among the pioneers who initiated the field of orthogeriatric care in late 1950s.^5^The term orthogeriatric care was formally introduced in 1977, and the first study reporting such type of collaboration was published in mid-eighties.^6^It is reported that the presence of geriatricians in orthopedic surgery wards can improve patients outcomes including; activities of daily living, number of medical complications, re-admissions, in-hospital mortality, discharges to nursing homes, and long-term restitution.^7-11^There are several empirical studies that support better outcomes for hip surgery when elderly have been treated by a team of orthopedic surgeons and geriatricians.^12-15^

Hospital stay is known to be the most costly component of acute care for hip fracture. Length of stay (LOS) is measured as the hospital’s level of efficiency in management of patients with hip fracture.^3^Several studies have shown that reducing LOS can lead to reduction in total health care cost.^16,17^However, it is also known that too short of stay in hospital during the acute phase of hip fracture treatment may result in worse patient outcomes.^18^

The aim of this study is to evaluate the efficiency of multidisciplinary geriatric in-hospital intervention on patient outcomes, among a sample of elderly patients with hip fracture.

## Methods

**Design**

This was a retrospective study with two historical cohorts. The first cohort served as control group. The patients in the second cohort constituted the intervention group.

**Participants**

The medical record of 495 patients age 65 years or older, with a primary diagnosis of hip fracture (femoral neck, intertrochanteric), who were consecutively admitted to the Orthopedic Department of Aarhus University Hospital, in two periods between July 1st to December 31st, in 2000 (control group), and July 1st to December 31st, in 2003 (intervention group), were reviewed. While the intervention group received extended treatment by a team of geriatricians and orthopedic surgeons, the control group only received traditional rehabilitation in the orthopedic ward. All patients in both groups were admitted from the same area of the city, and those who were transferred to other hospitals, after the surgery, were excluded from the study.

**Intervention**

The two year planning, development, and implementation of the geriatric multidisciplinary team in Aarhus University Hospital, Orthopedic surgery ward, ended in 2003. This explains the gap in patient recruitment for control and the intervention group during the period between year 2000 and 2003.

A geriatric team (GO-team), consisting of a geriatrician, a physiotherapist and a nurse with geriatric expertise, provided full-time geriatric and orthopedic care during daytime on weekdays. On weekends or off days the GO-team usually was not available. The GO-team included the following components in the care of their patients: conducting initial physical and mental screening and evaluation; providing continuity of care including geriatric focused care, and early discharge planning. Geriatricians and orthopedic care providers shared responsibility for patients’ care throughout the hospitalization period. The GO-team conducted rounds with the staff in the orthopedic ward and provided written report in patients’ medical charts/records.

One of the main goals of the GO-team was to minimize time to surgery and to avoid risk of iatrogenic illness. Information on the physical and mental status of the patient before hospitalization was obtained from the homecare system, the general practitioner, and the relatives, in order to elucidate complex social and medical conditions. To prevent future “fall-episodes”, patients were evaluated for diagnosis of osteoporosis and subsequently applied preventive measures. Of measures that GO-team took to prevent delirium or iatrogenic illness were to facilitate early discharge from the orthopedic ward; i.e., one or two days after the surgery. Patients who lived at home or lived at handicap-friendly housing were either: 1) escorted directly from the orthopedic ward to their homes with follow-up home-visits by a physiotherapist, or 2) transferred to a geriatric rehabilitation unit with multidisciplinary expertise. The choice between the two options depended on required personal and medical assistance. After the surgery, patients in the intervention group received training in ways to increase their mobility in activities of daily living (ADL). The GO-team also supervised patients’ nutritional in-take and managed patients’ concurrent medical problems including pain management and fluid therapy, which are among the most important factors in management of orthopedic elderly patients.

Patients admitted from nursing homes were returned to the nursing home directly from the orthopedic ward, without receiving geriatric follow-up since these patients had regular access to medical care by general practitioners, and the 24-hour staff in the nursing homes was close to the residents.

**Data collection and main outcome measurement **

Data regarding gender, age, housing, cause of fall, osteoporosis prophylaxis and iron treatment before and after admission, time to surgery, number of blood transfusions, and re-operations were collected from patient records. B-hemoglobin measurements at the time of the admission and three to six months post admission were obtained from the hospital electronic laboratory information system. Variations in the levels of hemoglobin between the time of admission and three to six months post admission, changes in discharge destination, three-month readmission rate, readmission rate within three to six months, new fractures within two years, in-hospital mortality and three-month mortality were collected as patient outcomes.

Length of stay (LOS) was the main outcome variable measuring hospital efficiency. Measurements for osteoporosis prophylaxis were an additional process outcome on hospital efficiency (Table 1). Data on LOS, readmissions, reoperations, and deaths were obtained from the Danish Health Data Bank (“Sundhedsdatabanken”). Information regarding new fractures was obtained from the emergency department, out-patient clinic, and from the Patient Registration System (Figure 1). All data were collected retrospectively by a nurse specialist.

**Table:** Table 1:**Baseline characteristics**

	Intervention group n = 233(%)	Control group n = 262 (%)
Age		
Mean (sd)	82.6 (7.83)	82.1 (7.73)
65-85 years old	137 (59)	164 (63)
> 85-max years old	96 (41)	98 (37)
Gender		
Female	180 (78)	211 (80)
Male	52 (22)	51 (20)
Housing up to admission		
Living at home	147 (63)	149 (59)
Handicap-friendly housing	37 (16)	40 (16)
Nursing home	48 (21)	65 (26)
Cause of fall		
Accident	114 (49)	69 (26)*
Medical cause	42 (18)	26 (10)
Unspecified	77 (33)	167 (64)
Blood tests on admission		
Mean hemoglobin (sd)	7.7 (0.93)	7.5 (0.90)*
Mean sodium (sd)	137 (4.5)	139 (4.3)*
Mean potassium (sd)	3.82 (0.51)	3.95 (0.51)*
Treatment up to admission		
Calcium with vitamin D	45 (19)	21 (8)*
Bisphosphonate	21 (9)	10 (4)*
Iron	32 (15)	18 (7)*
Time to surgery in days		
Mean (sd)	0.88 (0.79)	0.70 (0.98)*

* p >≤ 0.05

**Figure 1: Design of historical study of elderly patients with hip fracture divided into orthogeriatric intervention group and orthopedic control group:**
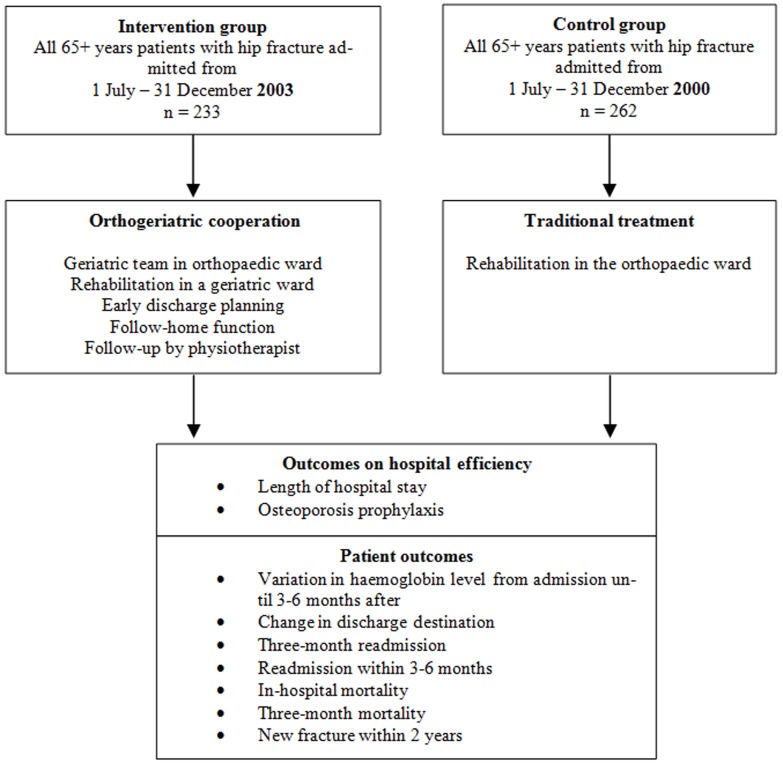


**Statistical analysis**

The differences in clinical characteristics between the two groups were tested by χ2 tests for proportions of categorical variables and presented in percentages and p-values with a significance level of 5%. In calculating differences in the continuous baseline characteristics, unpaired t-tests were used after a test of equal standard deviation in the two groups. LOS was tested for normal distribution and a logarithmic transformation was made to perform a multiple linear regression. Logistic regression was used for variables with dichotomized outcomes and adjusted for relevant potential effect variables. Then the residual-assumption in the regression model was checked and accepted. In analyzing the incidence of new fractures within two years, “lost to follow-up” was taken into account.^19^Data were analyzed in Intercooled Stata.^9,1^

## Results

Complete data for nearly all patients in the study was obtained. Only six patient's records for the period between 2000 until 2003 were missing. Both groups of patients had access to the same hospital resources and had fairly similar characteristics at the time of admission except that the intervention group had better baseline indicators with regard to osteoporosis prophylaxis and treatment of anemia, and also a higher hemoglobin level. Causes of falls differed between the groups but were not clearly indicated in the patient’s chart. A large proportion of the patients could not recall the fall episode. Potassium and sodium levels at baseline were lower in the intervention group (Table 1).

The total LOS at hospital in 2003 was reduced by the multidisciplinary geriatric intervention (Table 2). The overall median LOS was reduced from 15 to 13 days. In patients admitted from their homes, median LOS was reduced from 20 to15 days and in patients admitted from nursing homes, from five to three days. Patients admitted from handicap-friendly housing had one extra day of hospitalization, from 16 to 17 median days.

**Table:** Table 2:**Outcomes of the geriatric intervention on length of stay, variation in hemoglobin level, change in discharge destination, readmission, new fracture, and mortality**

			Geriatric Intervention	
Outcomes	Intervention n = 233	Control n = 262	Crude estimate(95% CI)^a^	Adjusted estimate ^b^ (95% CI)
LOS (median days)	13	15	β ^c ^: 0.92 (0.87; 0.97)	β : 0.67 (0.58; 0.79)
B-hemoglobin variation ^d ^(mean) mmol/l	0.23	0.19	β: 0.04 (-0.20; 0.28)	β : 0.04 (-0.19; 0.27)
Change in discharge destination (%)	35 (16)	52 (21)	OR ^e^ : 0.75 (0.47; 1.20)	OR: 0.93 (0.52; 1.65)
Three-month readmission (%)	30 (13)	32 (12)	OR: 1.13 (0.78; 1.64)	OR: 1.09 (0.71; 1.67)
Re-operation (%)	21 (9)	8 (3)	OR: 3.75 (1.52; 9.27)	OR: 3.87 (1.45; 10.3)
Readmission within three-six month (%)	63 (27)	68 (26)	OR: 1.17 (0.79; 1.73)	OR: 1.10 (0.70; 1.73)
Re-operation (%)	10 (4	6 (2)	OR: 1.75 (0.54; 5.66)	OR: 2.32 (0.56; 9.59)
**New fracture**				
Within 24 month (%) (n=157/ 172)	18 (12)	25 (15)	IRR ^f^ : 0.72 (0.39; 1.32)^*^	
In-hospital mortality (%)	18 (8)	16 (6)	OR: 1.29 (0.64; 2.59)	OR: 1.07 (0.49; 2.32)
Three-month mortality				
- All patients (%)	38 (16)	39 (15)	OR: 1.11 (0.69; 1.82)	OR: 1.25 (0.71; 2.20)
- Patients from homes and handicap- friendly housing (%)	19 (10)	26 (13)	OR: 0.75 (0.40; 1.41)	OR: 0.75 (0.35; 1.60)
- Nursing home residents (%)	20 (41)	15 (21)	OR: 2.67 (1.19; 5.96)	OR: 2.37 (0.99; 5.67)

a) CI = Confidence interval b) Adjusted for gender, age, housing, cause of fall, osteoporosis and iron treatment before hospitalization, b-hemoglobin, plasma potassium and plasma sodium at admission c) Coefficient (multiple linear regressions) d) Variation in b-hemoglobin at admission until three to six month after hip fracture. e) Odds ratio (logistic regression) f) Incidence rate ratio *) Estimate adjusted “lost to follow-up”

During the hospitalization, more patients in the intervention group were treated for osteoporosis after hip fracture. Of the patients who were not already in treatment with calcium/vitamin-D, 67 % of the patients began this treatment vs. 2% in the control group. In the intervention group treatment with bisphosphonate was initiated in 10% of the patients vs. 1% in the control group.

Hemoglobin levels increased significantly in both groups between the time of admission and three to six months post discharge, but there was no significant difference between the groups (Table 2). Due to low postoperative hemoglobin level, iron therapy was commenced more often in the intervention group (OR = 2.97 [95% CI: 1.95; 4.50]) compared to the control group. There was no observed difference in the number of blood transfusions during hospitalization in both groups. Geriatric intervention was not associated with more elderly remaining at homes after discharge (OR = 0.93 [95% CI: 0.52; 1.65]).

With regards toreadmissions, no significant difference was observed between the two groups even though 31 patients from the intervention group had a reoperation within six months of the fracture, primarily due to collapse of the hip fracture and chafing screws, compared to 14 patients in the control group (p<0.05). Fifty eight percent of the three-month readmitted cases in the intervention group were due to causes not related to the fracture, versus 48 % in the control group. In the geriatric intervention group, the risk of new fractures within two years of hospital discharge was not reduced significantly (incidence rate ratio = 0.72 [95% CI: 0.39; 1.32])(Table 2)

In-hospital mortality rate was 8% in the intervention group vs. 6% in the control group (OR=1.07 [95% CI: 0.49; 2.32]). Three-month mortality was 16% in the intervention group and 15% in the control group (OR= 1.25 [95% CI: 0.71; 2.20]). Three-month mortality was associated with housing at the time of admission (OR=1.45 [95% CI: 1.20; 1.74]). Residents from nursing homes in the intervention group had slightly over twice the odds of dying within three months compared to residents from the nursing home in the control group (OR=2.37 [95% CI: 0.99; 5.67]) (Table 2). Causes of death in intervention group residents include postoperative pulmonary edema, stroke, cancer, pneumonia, decompensated cardiac disease, chronic obstructive pulmonary disease, acute myocardial infarction, renal failure, and cirrhosis.

## Discussion

In the current study, we were able to show that the reduced LOS did not change the overall patient outcomes, while also not adding additional impairments. However, multidisciplinary geriatric intervention was not able to improve patient outcomes in conjunction with the reduced LOS.

Length of stay in patient with hip fracture varies greatly among countries from 6.6 to 32.5 days.^11^In a previous Danish study of accelerated continuity of care in elderly patients with hip fracture, the median LOS was reduced from 21 to 11 days. The rate of in-hospital mortality in that study was 16% compared to 8% in our study.^20^Examining hospital policies in respect to patient admission and LOS, for the period between 2000 and 2003, in Denmark, we were not able to find any major changes in those polices that could have explained shorter LOS among the intervention group in our study. However, it is reasonable to suggest that improved cooperation with the home care system, accelerated rehabilitation after hip fracture, improvements in the administration of anesthesia and types of aesthetic drugs, as well as surgical procedures, and a general trend in hospitals to shorten hospital stays may have contributed to the shorter LOS in the intervention group.^21^

Our findings also point out that in-hospital geriatric interventions significantly improved the rate of osteoporosis prophylaxis use but still many patients were discharged without treatment or referral for further examinations which is consistent with findings from previous studies.^22,23^Furthermore, hemoglobin values for the time between the admissions and three to six month follow-up did not improve in either of the groups. We speculate that this was partly due to GO-team lack of access and control over treatment of anemic patients after their discharge from the hospital.

Hospital readmission in elderly population is common and rate of hospital readmissions in this population is quite high. Almost half of the readmissions occur within the first three months of hospital discharge.^24^In our study the geriatric intervention did not reduce readmissions incidence in patients with hip fractures. This may be explained by the higher incidence of reoperations due to other causes in the intervention group. Although we do not rule out the possibility that early mobilization of patients in the intervention group may have contributed to a higher number of reoperations. Unfortunately, neither the type of fracture nor the type and methods of surgery with classes of implants were recorded in the patient’s medical chart. Therefore, we were unable to control for their association with high incidence of reoperations in our study. Analysis of data from the Danish database of Hip Fractures in The National Indicator Project may shed some light on this issue.^25^

Empirical evidence demonstrates that reduction in delay of surgery decreases mortality.^26^In our study, which also included frail elderly patients, time to surgery was longer in the intervention group. This could explain why we were unable to report reduction for in-hospital mortality rate or reduction in three-month mortality rate, despite the implementation of geriatric intervention. Among patients who were admitted from their homes or from handicap-friendly housing, and were followed after discharge, there was a trend towards reduced three-month mortality (Table 2). Unfortunately, data was not collected to study the association between admission during off hours and weekends -when geriatric intervention was not available-, with readmissions and post surgery mortality.

Negative patient outcomes in early discharges, often named “quicker but sicker”, seem to be relevant in the frail patients from nursing home.^11^In 2003, LOS for patients who resided in nursing homes was on average three day and they rarely were followed by the geriatric team after hospital discharge. This was unlike hip fracture patients who reside in homes or handicap-friendly housing who received follow-up care from the geriatric team. Furthermore, patients from the nursing homes are generally frailer than elderly patients living at home or in handicap-friendly housing. It seems that, the nursing home residents may have needed more comprehensive care during the first weeks of hospital discharge, even though they were living in nursing homes with the staff nearby and easy access to the general practitioner. The nursing home residents comprised 23% of our study sample and the estimate on the association with mortality was uncertain due to obtaining wide confidence interval. 

 The major limitation of this study had to do with the use of historical cohorts (control and intervention groups) that were recruited in the study from two different time periods, rather than two concurrent groups. However, the power of the sample was statistically high, indicated by the narrow confidence intervals in the tests of associations between the predictor and outcome variables.

In conclusion, our study indicates that a combined multidisciplinary geriatric and orthopedic surgical intervention in elderly patients with hip fracture may be associated with a reduction in length of hospital stay without negatively affecting major patient outcomes. The study suggests that the concept should be further developed and combined with other measures aimed at accelerating patient recovery time during the post surgery particularly among the frail elderly living in nursing homes. Future studies are also needed to replicate and verify the treatment efficacy of GO-team intervention not only in elderly patients with hip fractures but with other fractures as well. These studies should also include data on comorbidity to ensure better comparability between groups.
